# Biomarker-Based ABC-AF Risk Scores for Personalized Treatment to Reduce Stroke or Death in Atrial Fibrillation: A Registry-Based, Multicenter, Randomized, Controlled Study

**DOI:** 10.1161/CIRCULATIONAHA.125.076725

**Published:** 2025-08-30

**Authors:** Jonas Oldgren, Ziad Hijazi, Håkan Arheden, Anna Björkenheim, Viveka Frykman, Magnus Janzon, Annica Ravn-Fischer, Henrik Renlund, Anders Själander, Torbjörn Åkerfeldt, Lars Wallentin

**Affiliations:** 1 Uppsala Clinical Research Center (J.O., H.R., L.W.), Uppsala University, Uppsala, Sweden.; 2 Department of Medical Sciences (J.O., Z.H., L.W., T.Å.), Uppsala University, Uppsala, Sweden.; 3 Department of Clinical Sciences Lund, Lund University, Lund, Sweden (H.A.).; 4 Department of Clinical Physiology, Skåne University Hospital, Lund, Sweden (H.A.).; 5 Department of Cardiology, Örebro University, Örebro, Sweden (A.B.).; 6 Department of Clinical Sciences, Danderyd University Hospital, Karolinska Institutet, Stockholm, Sweden (V.F.).; 7 Departments of Cardiology and Health, Medicine, and Caring Sciences, Linköping University, Linköping, Sweden (M.J.).; 8 Department of Cardiology, Sahlgrenska University Hospital, and Institute of Medicine, Department of Molecular and Clinical Medicine, Sahlgrenska Academy University of Gothenburg, Gothenburg, Sweden (A.R.-F.).; 9 Department of Public Health and Clinical Medicine, Umeå University, Umeå, Sweden (A.S.).

**Keywords:** atrial fibrillation, biomarkers, intracranial hemorrhages, risk factors, stroke

## Abstract

**BACKGROUND:**

The clinical use of risk scores to guide treatment decisions and improve clinical outcomes has rarely been prospectively evaluated. This study aimed to evaluate whether a biomarker-based ABC-AF risk score–guided multidimensional treatment strategy improves long-term outcomes in patients with AF.

**METHODS:**

The multicenter, registry-based, randomized, controlled, open-label study enrolled adults with AF. In the ABC-AF strategy arm, the investigator was informed of each individual’s ABC-AF score risks for stroke and bleeding, which were used as decision support to tailor treatment recommendations, including preference for type of direct oral anticoagulant treatment. In the standard of care arm, patient management was at the discretion of the investigator. Primary outcome was a composite of stroke or death. Secondary outcomes included stroke, death, major bleeding events, and their composite outcome.

**RESULTS:**

The intention-to-treat population comprised 3933 patients with a median age of 73.7 years; 33.6% were women, 51.3% had paroxysmal AF, 11.2% had a previous stroke or transient ischemic attack, and 85.7% had oral anticoagulant treatment. After randomization, 97.8% in the ABC-AF strategy arm and 92.6% in the standard of care arm received OACs (*P*<0.0001). Enrollment was prematurely terminated owing to safety concerns with a trend toward higher mortality in patients with CHA_2_DS_2_-VASc scores of ≥3, and the study was therefore underpowered for its primary objective. Over a median follow-up of 2.6 years, 175 primary events (3.18/100 patient-years [100PY]) occurred in the ABC-AF strategy and 148 (2.67/100PY) in the standard of care arm (hazard ratio [HR], 1.19 [95% CI, 0.96–1.48]; *P*=0.12). Major bleeding events were 152 (2.82/100PY) versus 141 (2.61/100PY; HR, 1.08 [95% CI, 0.86–1.36]; *P*=0.50), stroke 48 (0.87/100PY) versus 41 (0.74/100PY; HR, 1.18 [95% CI, 0.78–1.79]; *P*=0.44), death 136 (2.44/100PY) versus 113 (2.02/100PY; HR, 1.21 [95% CI, 0.94–1.55]; *P*=0.13), and rates of composite stroke, death, or major bleeding 277 (5.21/100PY) versus 244 (4.55/100PY; HR, 1.14 [95% CI, 0.96–1.36]; *P*=0.13). Primary outcome results were similar across ABC-AF score subgroups (interaction *P*=0.98).

**CONCLUSIONS:**

The individually tailored multidimensional treatment strategy, based on ABC-AF risk scores, did not improve clinical outcomes compared with usual guideline-based care in patients with AF. The results emphasize the need for prospective testing of the use of risk stratification and precision medicine tools in different clinical settings before implementation in routine care.

**REGISTRATION:**

URL: https://www.clinicaltrials.gov; Unique identifier: NCT03753490.

**Clinical Perspective**

**What Is New?**

- The clinical use of risk scores to guide treatment decisions and improve clinical outcomes in cardiovascular diseases has rarely been prospectively evaluated.
- This pragmatic, multicenter, registry-based, randomized clinical study evaluated whether a biomarker-based ABC-AF risk score–guided multidimensional treatment strategy improves clinical outcomes in patients with atrial fibrillation compared with usual guideline-based care.
- The individually tailored multidimensional risk–based treatment strategy was not associated with any improvements in clinical outcomes in patients with atrial fibrillation at a low to moderate risk of stroke during oral anticoagulant treatment.

**What Are the Clinical Implications?**

- The use of all types of risk stratification and precision medicine tools should be prospectively tested in different clinical settings before their implementation in routine clinical care.
- Precise and quantitative risk scores may still be considered for identification of patients with atrial fibrillation at high risk for stroke or bleeding in other settings.

**Editorial, see p 1470**

Atrial fibrillation (AF) is the most common cardiac arrhythmia, and the prevalence is higher at an older age and with concomitant cardiovascular disease. Patients with AF have an increased risk of stroke, heart failure, and death. Current guidelines recommend a multifactorial approach to reduce the risk for complications in patients with AF.^1–4^ Treatment with oral anticoagulants (OACs), nowadays usually with direct OACs (DOACs), substantially reduces the risk of stroke in patients with AF but is associated with an increased risk of major bleeding, whereas the risk for intracranial bleeding is substantially lower with DOACs compared with warfarin.^5–9^ Both the European and US guidelines recommend a risk-based approach to decisions on anticoagulation and other treatments for stroke prevention in patients with AF.^3,4^ Available OACs have different profiles concerning the balance between efficacy and safety, which might be used when selecting the most appropriate OAC in relation to risk in the individual patient.^5–9^

In recent years, novel biomarker-based risk scores for stroke and bleeding in patients with AF have been developed, repeatedly validated, and calibrated in several large geographically diverse populations across all inhabited continents. The biomarker-based ABC-AF-stroke score (age, biomarkers [NT-proBNP (N-terminal pro-B-type natriuretic peptide) and hs-troponin T] and clinical history of stroke/transient ischemic attack [TIA]) provides a quantitative estimate of the risk of stroke as well during treatment with DOACs or warfarin as without any OACs.^10–14^ Similarly, the biomarker-based ABC-AF-bleeding score (age, biomarkers [GDF-15 (growth differentiation factor 15), hemoglobin, and hs-troponin T] and clinical history of bleeding) provides a quantitative estimate of the risk of major bleeding during DOAC or warfarin treatment,^10,11,15^ which can be balanced against the quantitative risk of stroke.^16^ The implementation of these biomarker measurements in laboratories and the automatic calculation of the risk scores in electronic health records or smartphones provides a novel basis for individual treatment recommendations on the basis of more precise estimates of risks and continuous rather than dichotomized variables.

The clinical use of risk scores to guide treatment decisions and improve clinical outcomes in AF has rarely been prospectively evaluated. We therefore performed a prospective registry-based randomized clinical trial to evaluate whether the tailoring of multidimensional treatment recommendations, based on the patient’s ABC-AF risk score results, improves clinical outcomes compared with usual guideline-based care in patients with AF.

## Methods

The primary study objective was to evaluate whether personalized treatment recommendations based on quantitative estimates of the risk of stroke and bleeding by the ABC-AF biomarker-based risk scores reduce the occurrence of the composite outcome of stroke or death without increasing the risk of bleeding in patients with AF. Longer-term follow-up of the ABC-AF study population is ongoing. Deidentified data will be made available upon reasonable request after publication of the long-term results.

### Study Design

The detailed study design has been published.^17^ In brief, eligible patients for this multicenter, prospective, registry-based, randomized, controlled, open-label study were adults with a diagnosis of AF, including newly or previously diagnosed, with or without current OAC treatment, with a limited number of exclusion criteria: contraindication of any OAC according to the summary of product characteristics; indication for OAC treatment beyond AF (eg, venous thromboembolism and/or mechanical heart valve prosthesis); currently on treatment with a DOAC and not eligible/not a candidate for a change of DOAC drug (eg, because of drug–drug interactions); concomitant dual antiplatelet treatment; acute coronary syndrome within 30 days; participation in an antithrombotic pharmaceutical trial; planned AF ablation or AF surgery; hemoglobin <90 g/L; and patients who, in the opinion of the investigator, cannot or will not comply with the requirements of the protocol.

Patients in both treatment arms were recruited, randomized, and managed by the same investigators at each site. After written informed consent, baseline characteristics and treatments were registered online in the Swedish national quality register for AF (AURICULA AF). Patients were randomized 1:1 to either an ABC-AF risk score–guided treatment strategy or to standard of care. Randomization was stratified by study site and performed within a study-specific module in the AURICULA AF registry. Plasma samples for biomarker analyses were obtained at randomization from patients in the ABC-AF strategy arm and sent for direct measurement. When feasible, plasma samples from all patients were aliquoted and locally stored frozen at −80°C until the end of the study. In the ABC-AF strategy arm, the biomarker analyses for ABC-AF risk scores were performed within 2 working days. The ABC-AF risk scores were automatically calculated based on the ABC-AF-stroke score variables (age, biomarkers [NT-proBNP and hs-troponin T], and clinical history of stroke) and the ABC-AF-bleeding score variables (age, biomarkers [GDF-15, hemoglobin, and hs-troponin T], and clinical history of major bleeding). Patients were categorized into one of 6 risk groups, and the investigator received a visual presentation of the ABC-AF risk scores for stroke and bleeding, along with specific treatment recommendations tailored to each individual patient based on the estimated stroke and bleeding risks, for patients deemed suitable for stroke prevention treatment, as detailed in the Supplemental Methods. Based on this information, the investigator decided on medical treatments and other interventions for the patients in the ABC-AF strategy arm.

Patients in the control arm were managed in accordance with local practices, and national and international guidelines at the discretion of the investigator, without measurements of any ABC-AF risk score biomarkers and without any study-specific individual treatment recommendations.

### Study Outcomes

The primary outcome of the study was a composite of stroke or death of all causes. Secondary outcomes included a composite of stroke, death, or major bleeding; individual components of the primary outcome; myocardial infarction; and hospitalization for heart failure. Major bleeding was the primary safety outcome, with subdivisions of intracranial or extracranial major bleeding events.

Data on primary and secondary study outcomes were retrieved bimonthly from the Swedish Population Register throughout the study period, which includes data on vital status for all Swedish residents, and every third month from the mandatory National Patient Register, covering all in-patient care at all Swedish hospitals, and the Cause of Death Register, with the last two hosted by the National Board of Health and Welfare. To ensure completeness of outcome data while taking into account delayed reporting of health care episodes from Swedish hospitals to the National Patient Register, data for the present analysis were retrieved from this register 18 months after recruitment of the last included patient.

### Study Treatments

Information on medications at study inclusion and after randomization was collected in the AURICULA AF registry, except for proton pump inhibitors, which are not captured in this registry. Information on pharmacological treatments during long-term follow-up was assessed by data obtained from the National Drug Prescription Register, which records all drug dispensation in Sweden (National Board of Health and Welfare).

### Sample Size and Statistics

The annual rate of the primary composite outcome of stroke or death was anticipated to be 8.0% in the standard of care arm based on previous experience of event rates in the corresponding Swedish registry population.^18^ To provide a statistical power of 80% for a 15% relative reduction of the primary outcome, a total of 1191 events were needed to test the primary hypothesis, and 6500 patients were needed to generate this number of events. A negligible attrition rate was expected, as the components of the primary composite of stroke and death have extremely high coverage in the Swedish mandatory registries used for this study.

The primary composite of stroke or death was tested in the intention-to-treat population using a log-rank test, with a Cox regression model used as sensitivity analysis and to evaluate homogeneity in findings in the prespecified subgroups sex, age category (<65, 65–74, and ≥75 years of age), previous stroke or TIA, and CHA_2_DS_2_-VASc (congestive heart failure, hypertension, age, diabetes, previous stroke/transient ischemic attack, vascular disease, age, sex) category (women <3 and men <2 points versus women ≥3 and men ≥2 points). The secondary outcomes were also analyzed with a log-rank test alongside Cox regression models for sensitivity. The significance level was 5%, two sided.

In a post hoc exploratory analysis, the primary composite outcome of stroke or death was analyzed with a Cox regression model in the prespecified categories according to ABC-AF-stroke and ABC-AF-bleeding risks, including testing of interactions between ABC-AF score groups and the effect of randomized strategy on outcomes.

McNemar and χ^2^ tests were used to determine differences in key treatments within the randomized groups before and after randomization and between the randomized groups after randomization, respectively. One-year persistence to key treatments is presented graphically.

All statistical analyses were performed using R software, version 4.5.0 (R Foundation for Statistical Computing, Vienna, Austria).

### Study Organization

The study was initiated by the investigators in the steering committee who were responsible for the design, conduct, and reporting of the trial. The study was performed by the Uppsala Clinical Research Center (Uppsala, Sweden),^19^ which was responsible for study and site coordination, the national AURICULA quality registry and study database, data management, and the planning and performance of the statistical analyses. The coordinating investigator had full access to all data in the study and takes responsibility for its integrity and the data analysis. Funding was obtained from the Swedish Research Council, the Swedish Heart-Lung Foundation, the Swedish Foundation for Strategic Research, and a grant, reagents, local laboratory analyses, and biobanking from Roche Diagnostics International Ltd. (Rotkreuz, Switzerland). The ABC-AF study was centrally approved by the Swedish Ethical Review Authority (Dnr 2017/386). All patients provided individual written informed consent for participation.

## Results

Enrollment started on October 25, 2018. The steering committee decided to terminate patient recruitment prematurely on May 12, 2023, and to analyze the results after 12 months of follow-up of the last enrolled patient. This decision was based on a recommendation from the data monitoring committee owing to safety concerns with a trend toward higher mortality in patients with CHA_2_DS_2_-VASc scores of 3 or higher in the ABC-AF strategy arm and the results of an informal assessment of futility for the efficacy of the ABC-AF risk score–guided treatment strategy. All randomized patients were followed until May 12, 2024, or until death, except for 3 patients who withdrew consent during the study, 1 in the ABC-AF strategy arm and 2 in the standard of care arm (Figure S1). The median follow-up was 2.6 years.

The intention-to-treat population enrolled at 37 Swedish sites comprised 3933 patients, of whom 33.6% were women; 1971 were randomized to the ABC-AF strategy and 1962 to the standard of care arm. The randomized groups were well balanced (Table 1). Median age was 73.7 (25th percentile, 66.8; 75th percentile, 78.8), with 18.7% 18 to 65 years of age and 46.6% ≥75 years of age. Body mass index was 26.9 (24.4;30.4) kg/m^2^, blood pressure was 130 (120;142) mm Hg systolic, and 80 (70;85) mm Hg diastolic, hemoglobin was 142 (132;151) g/L, and creatinine was 83 (71;96) μmol/L.

**Table 1.**
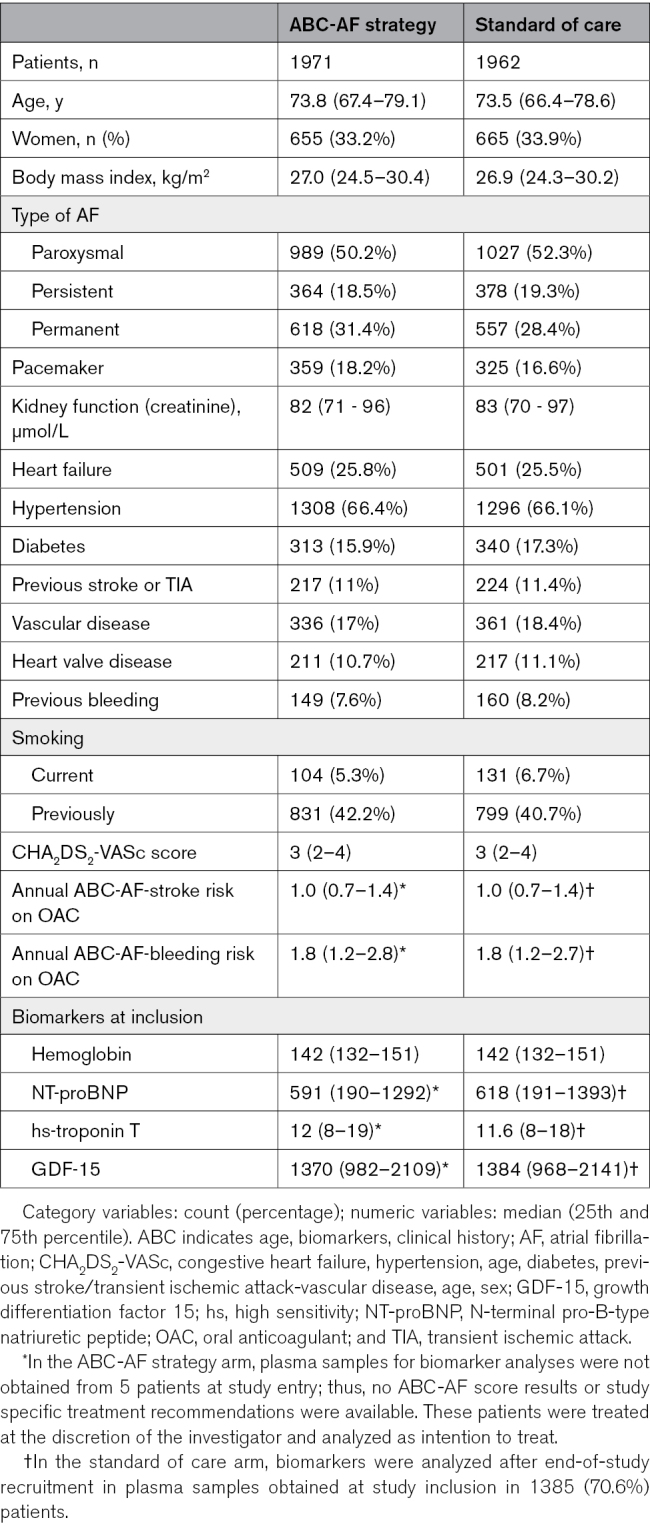
Baseline Characteristics

AF was paroxysmal in 51.3%, persistent in 18.9%, and permanent in 29.9% of the patients. A previous stroke or TIA was reported in 11.2% of the patients, 25.7% had heart failure, 66.2% had hypertension, 16.6% had diabetes, and 77.1% had high CHA_2_DS_2_-VASc scores (men, ≥2 and women, ≥3). A previous major bleeding event was reported in 7.9% of the patients, including 1.2% with previous intracranial bleeding.

The number of patients in the ABC-AF strategy arm within each of the 6 predefined ABC-AF-stroke and ABC-AF-bleeding risk categories and their corresponding treatment recommendations are depicted in Figure S2.

### Treatments Before and After Randomization

Treatments before and after randomization are displayed in Table 2. At study enrollment, the proportions of patients on different treatments were similar between the 2 randomized arms, with >85% on OAC treatment and <6% on antiplatelet treatment.

**Table 2.**
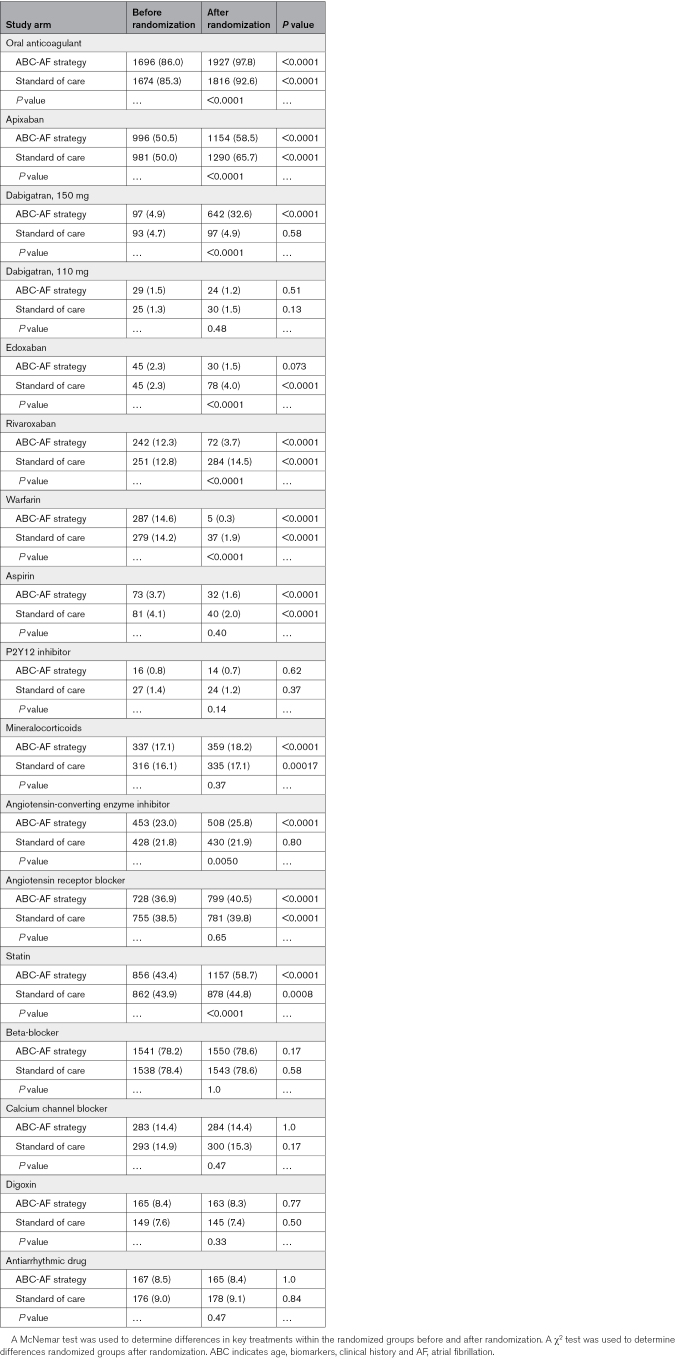
Pharmacological Treatments Before and After Randomization

**Figure 1.**
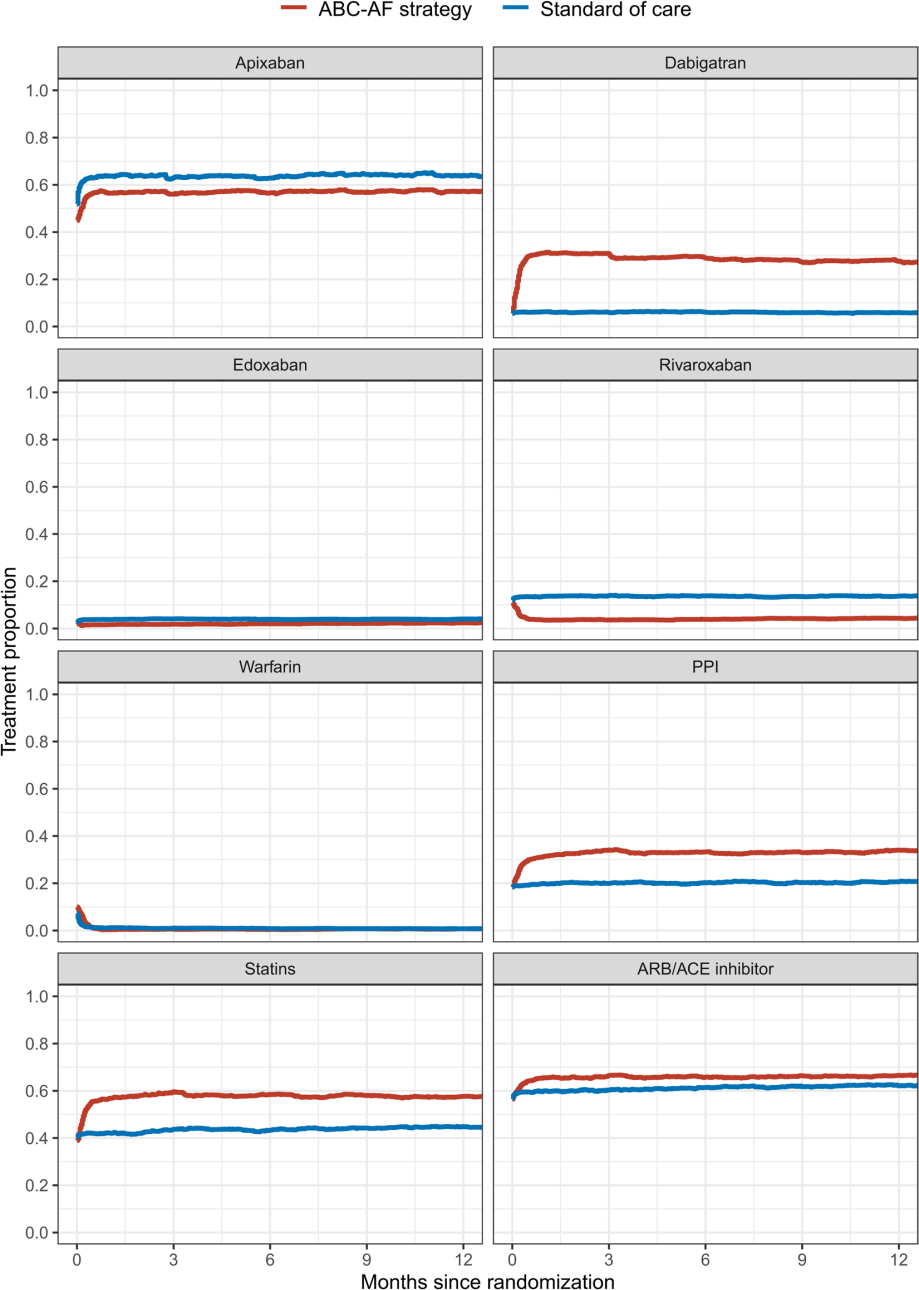
**One-year persistence to key medical treatments.** One-year persistence to key medical treatments in the ABC-AF strategy arm is shown in red and standard of care in blue. ACE indicates angiotensin-converting enzyme; ARB, angiotensin receptor blocker; and PPI, proton pump inhibitor.

After randomization, the proportion of patients receiving any OAC increased to 97.8% in the ABC-AF strategy arm and 92.6% in the standard of care arm (*P*<0.0001). In the ABC-AF strategy arm, the proportion of patients on apixaban was increased from 50.5% to 58.5%, and of those on dabigatran (150 mg), it was increased from 4.9% to 32.6%, whereas the proportion of those on rivaroxaban was reduced from 12.3% to 3.7%, and of those on warfarin, it was reduced from 14.6% to 0.3% (all *P*<0.0001). In the standard of care arm, use of apixaban was increased from 50.0% to 65.7%, edoxaban from 2.3% to 4.0%, and use of rivaroxaban was increased from 12.8% to 14.5%, whereas warfarin was reduced from 14.2% to 1.9% (all *P*<0.0001). The proportion on antiplatelet treatment was halved after randomization in both study arms. Statin treatment was increased from 43.4% to 58.7% in the ABC-AF strategy arm (*P*<0.0001) and from 43.9% to 44.8% in the standard of care arm (*P*=0.0008). Monitoring of long-term drug prescriptions showed a high persistence to these treatments (Figure 1). There were only minor changes in other tracked medications after randomization.

### Clinical Outcomes

**Figure 2.**
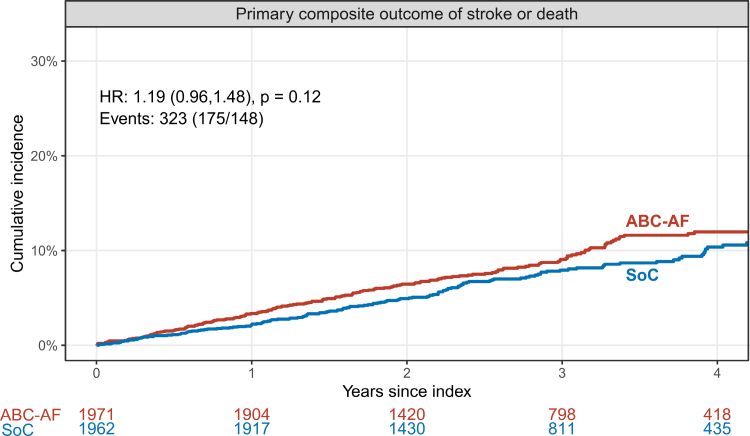
**Primary outcome.** The primary composite outcome was stroke or death. Shown is a Kaplan-Meier plot with the ABC-AF strategy arm in red and standard of care arm in blue. Shown is a Cox regression model with hazard ratios and 95% CI in parenthesis. HR indicates hazard ratio; and SoC, standard of care.

There were, in total, 323 primary composite outcome events of stroke or death (27% of the number anticipated in the power calculation), with 175 events (3.18/100 patient-years) in the ABC-AF strategy arm and 148 events (2.67/100 patient-years) in the standard of care arm (log-rank *P*=0.12; Table 3; Figure 2).

**Table 3.**
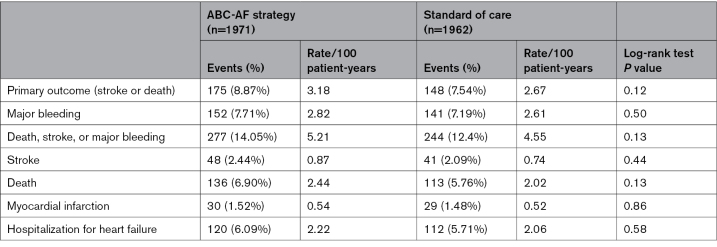
Primary and Secondary Outcomes

**Figure 3.**
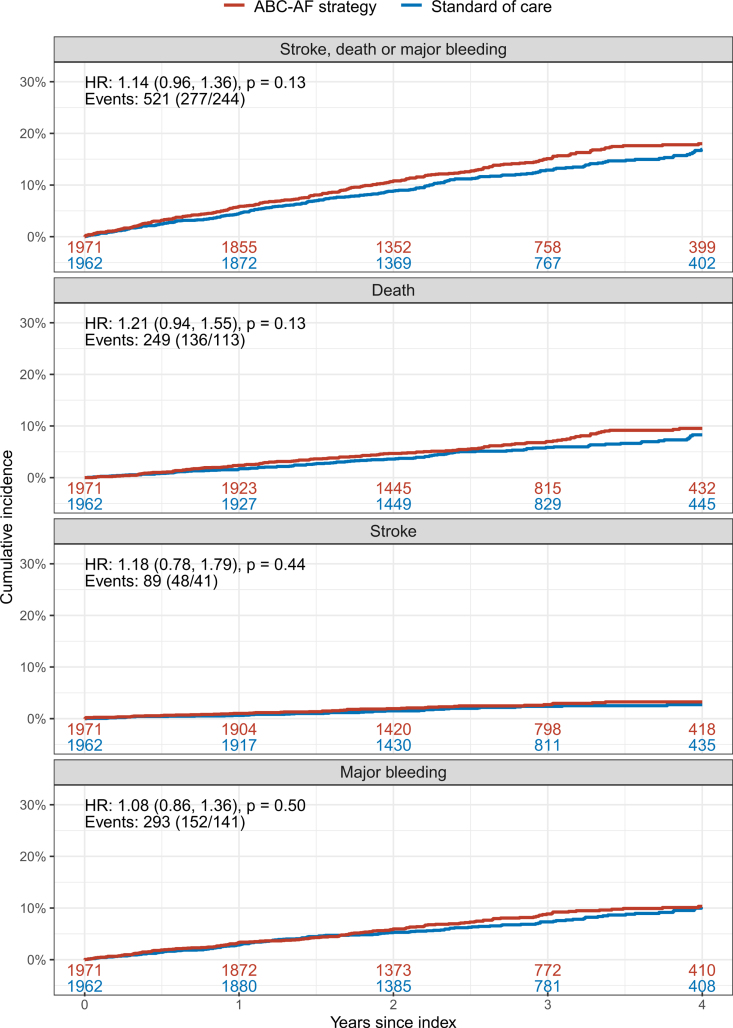
**Secondary outcomes.** Secondary outcomes were a composite of stroke, death, or major bleeding. Shown are Kaplan-Meier plots with the ABC-AF strategy arm in red and standard of care arm in blue. Shown are Cox regression models with hazard ratios and 95% CI in parentheses. HR indicates hazard ratio.

Major bleeding events were 293 in total, with rates of 2.82 per 100 patient-years in the ABC-AF strategy arm and 2.61 per 100 patient-years in the standard of care arm (*P*=0.50). Forty-three of the major bleeding events were intracranial hemorrhages, with rates of 0.38 per 100 patient-years in the ABC-AF strategy arm and 0.39 per 100 patient-years in the standard of care arm. There were 521 events of the secondary composite of stroke, death, or major bleeding events, with rates of 5.21 per 100 patient-years in the ABC-AF strategy arm and 4.55 per 100 patient-years in the standard of care arm (*P*=0.13). There were no significant differences in any of the other secondary outcomes (ie, stroke, death, myocardial infarction, or hospitalization for heart failure; Table 3; Figure 3).

**Figure 4.**
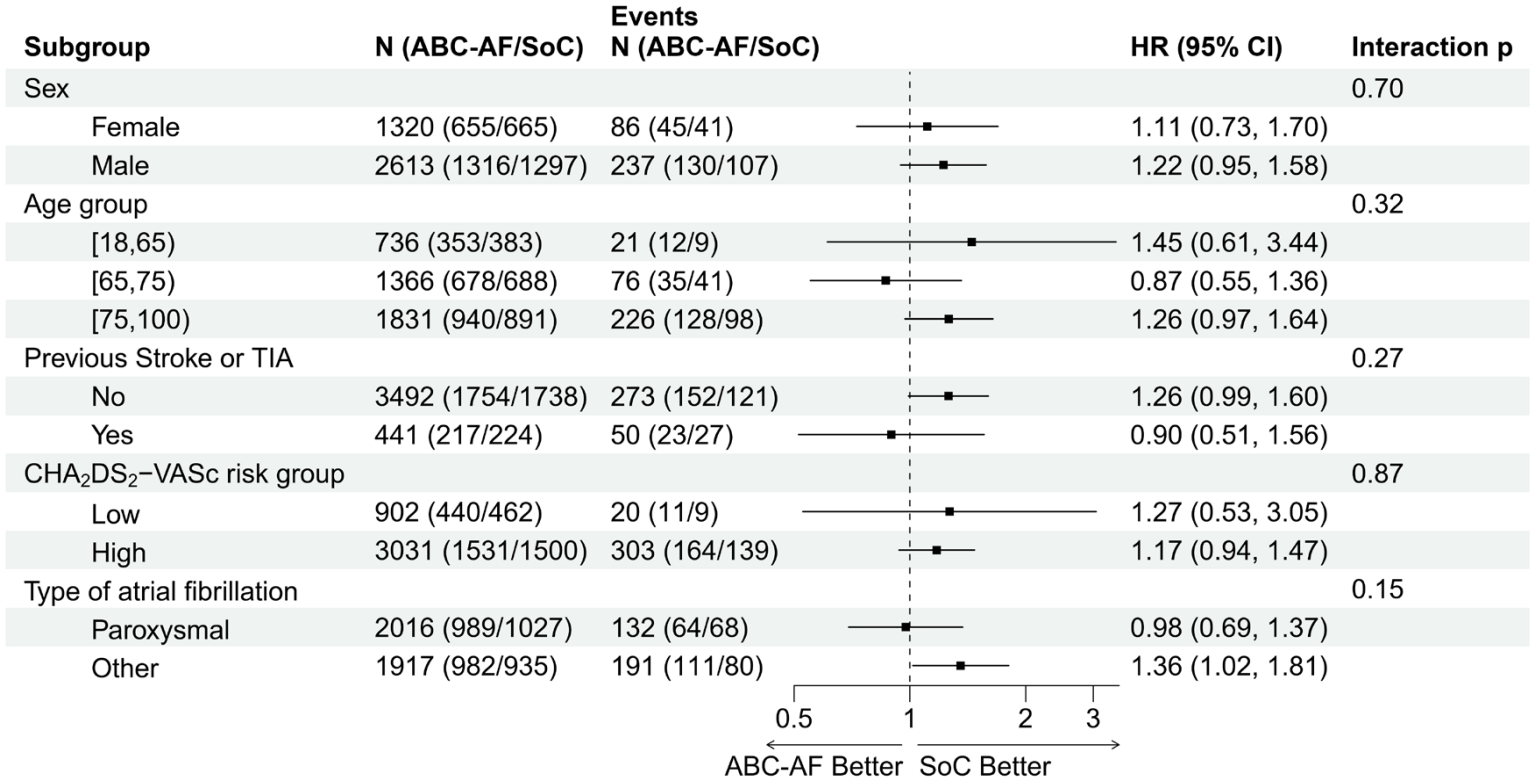
**Primary outcome in prespecified subgroups.** Shown is the primary composite outcome of stroke or death in prespecified subgroups: sex and age; previous stroke or transient ischemic attack; and CHA_2_DS_2_-VASc, with high defined as ≥2 points for men and ≥3 points for women. HR indicates hazard ratio; SoC, standard of care; and TIA, transient ischemic attack.

**Figure 5.**
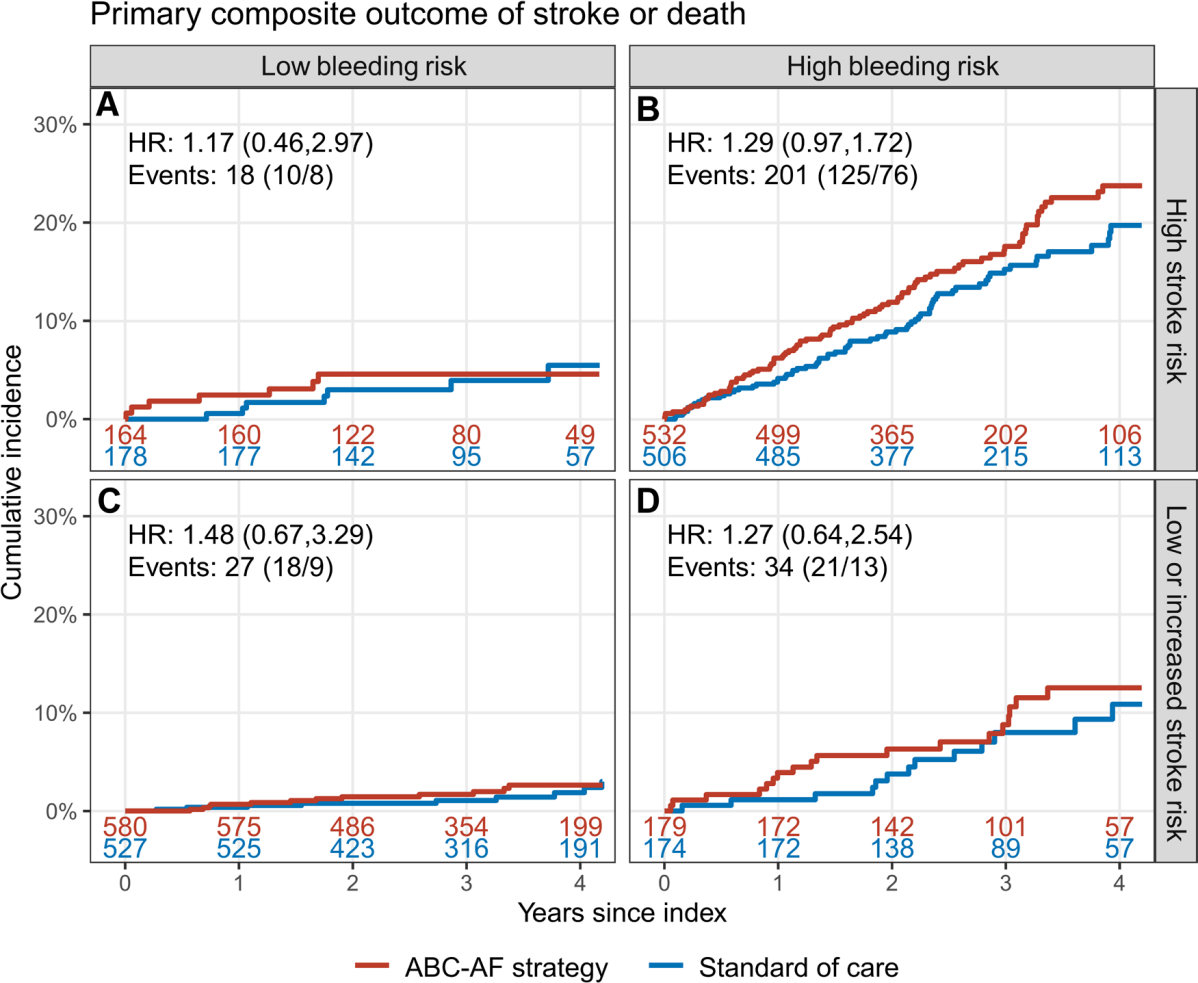
**Primary outcome in four risk subgroups by ABC-AF-stroke and ABC-AF-bleeding scores.** Shown is the primary composite outcome of stroke or death in 4 risk subgroups by ABC-AF-stroke and ABC-AF-bleeding scores. **A**, High stroke risk, low bleeding risk. **B**, High stroke risk, elevated bleeding risk. **C**, Low or elevated stroke risk, low bleeding risk. **D**, Low or elevated stroke risk, elevated bleeding risk. Kaplan-Meier plots show the ABC-AF strategy arm in red and standard of care arm in blue, comprising 2840 (of the 3933) patients enrolled at study centers with biobanked blood samples for patients randomized to the standard of care arm. Because of the limited number (n=65) of patients categorized as having low bleeding risk in this analysis, patients with low stroke risk and low bleeding risk (Supplemental Material, category 5) were analyzed with those with elevated stroke risk and low bleeding risk (category 3), and patients with low stroke risk and elevated bleeding risk (category 6) were analyzed with those with elevated stroke risk and elevated bleeding risk (category 4). *P*=0.98 for interaction (risk group×randomization). HR indicates hazard ratio.

There were no significant differences in the primary outcome of stroke or death in any of the prespecified subgroups: sex, age category (<65, 65–74, and ≥75 years of age), previous stroke or TIA, and CHA_2_DS_2_-VASc category (women <3 and men <2 points versus women ≥3 and men ≥2 points; Figure 4). The primary outcome results in the predefined ABC-AF risk subgroups, which constituted the basis for the treatment recommendations, are displayed in Figure 5. Results were similar across subgroups stratified by ABC-AF risks, with no significant interaction between risk subgroup and randomization.

## Discussion

This randomized clinical study found no benefit of individually tailored, multidimensional treatment recommendations on the basis of biomarker-derived quantitative estimates of individual risk of stroke and bleeding compared with usual guideline-based care in patients with AF. The changes in treatments and differences between treatment groups after randomization were modest, except concerning the type of DOAC. There were no significant interactions between sex, age, previous stroke or TIA, CHA_2_DS_2_-VASc or ABC-AF risk subgroups and randomization and the multidimensional treatment strategy on the primary outcome. The results emphasize the need for prospective testing of the use of risk stratification and precision medicine tools in different clinical settings before being implemented for tailoring treatment in routine clinical care.

There was a trend toward higher mortality at higher CHA_2_DS_2_-VASc scores in the ABC-AF strategy arm, which led to premature termination of recruitment and a shortening of follow-up until analysis of the results. The study was therefore underpowered for its primary objective, and the adverse trend might be exaggerated because of the early termination at the time of a warning signal. Still, it may be concluded that the individually tailored multidimensional medical treatment tested in this study is unlikely to benefit patients with AF treated with DOACs and other preventive measures recommended in international guidelines.

The inclusion criteria allowed enrollment of a broad population of patients with AF with relatively few exclusion criteria. Investigators were encouraged to approach and enroll older patients with AF and comorbidities associated with higher risk of stroke and death. Still, the risk profile of the ABC-AF study population was lower than in the COMBINE AF biomarker cohort with patients from the pivotal clinical trials of DOACs versus warfarin, as reflected by an ABC-AF-stroke score of 1.0 versus 1.2 and a median CHA_2_DS_2_-VASc of 3 versus 4, respectively.^14^ In addition, 51% of the ABC-AF study patients had paroxysmal AF compared with 23% in the pivotal studies of DOACs versus warfarin, which is also associated with lower risk of stroke or death. The substantially lower-than-anticipated primary outcome rates may, accordingly, reflect recruitment of patients at lower risk than planned. Moreover, the vast majority of patients were already on OAC treatment at study entry, and the use of, and persistence to, the initial DOAC prescription was very high in both study arms, limiting the possibility to determine an effect of the study intervention. The prestudy assumptions regarding differences in the balance between stroke prevention and bleeding risk among different DOAC drugs seem to have been overly optimistic. The acceptance and adherence to other specific recommendations on drug treatments and interventions for patients in the ABC-AF strategy arm were limited and may have had only a marginal or no effect on the risk of stroke, death, or major bleeding. Retrospectively, it appeared that a very high proportion of patients in the ABC-AF strategy and standard of care arms received adequate guideline-recommended OACs and other treatments to prevent stroke, death, heart failure, and myocardial infarction without inappropriately increasing the risk of major bleeding. It is possible that common treatment recommendations for patients in the ABC-AF strategy arm may have also indirectly impacted the investigators’ treatment decisions for patients in the standard of care arm. Therefore, both lower-than-aimed-for risk profiles and the high standards of care might explain the lower-than-expected event rates in this study population (stroke rate, 0.80%/year; death rate, 2.23%/year) than in the COMBINE biomarker cohort (1.3%/year and 3.62%/year, respectively).^14^ There are also conflicting study results on risks and benefits of switching of OACs, especially in older frail patients with AF. The randomized FRAIL-AF study indicated an increased risk of bleeding events without any benefit on thromboembolic outcomes after switching from a vitamin K antagonist to DOACs,^20^ whereas a recent analysis of the COMBINE-AF study revealed significant reductions in stroke or systemic embolism, fatal and intracranial bleeding, and death in frail, older, vitamin K antagonist–experienced patients who switched to standard-dose DOACs.^21^ The trend toward higher rates of all study outcomes in the ABC-AF strategy arm of the present study remains difficult to explain and might be attributable to chance, although it cannot be ruled out that the tested multidimensional treatment strategy was inferior to the standard of care.

The ABC-AF study was designed as a pragmatic study according to the PRECIS-2 (Pragmatic-Explanatory Continuum Indicator Summary 2) criteria,^22^ including use of the existing AURICULA-AF clinical quality registry for collection of baseline data, with the addition of an integrated study module that facilitated recording of informed consent and allowed randomization in the routine care setting.^23^ The broad eligibility criteria should have facilitated enrollment of patients representing usual clinical care. The study performance was highly cost-effective, as the follow-up by dedicated site personnel was limited to a single contact by phone after 3 months, whereas all clinical outcomes were, throughout the study, centrally obtained from the mandatory national registries. The events assessed as primary and secondary outcomes were considered the most relevant to the participants and to the evaluation of the use of new tools and treatment strategies in the care of patients with AF. The follow-up data are complete and reliable, as these public registries capture vital status and *International Classification of Diseases*–coded discharge diagnoses for all hospital admissions for an unlimited time,^24,25^ allowing ongoing longer-term follow-up of the study population.

Unfortunately, the use of precision medicine–based treatment strategies (whether informed by clinical data, laboratory data, multifactorial scoring systems, or artificial intelligence–based tools) has rarely been rigorously evaluated before their endorsement in treatment guidelines or implementation in clinical cardiovascular practice. A recent randomized study of cardiovascular screening with multiple preventive actions in >46 000 men found no effect on long-term mortality.^26^ In a randomized study of 1129 patients with AF, automated CHA_2_DS_2_-VASc–based decision support was not associated with any improvement in outcomes.^27^ A cluster-randomized study of 43 primary care clinics managing 8892 patients with AF found that CHA_2_DS_2_-VASc–based clinical decision support modestly improved guideline adherence to OAC therapy but without any differences in clinical outcomes.^28^ These results are in accordance with the findings of the present study. In another cluster-randomized study of 3628 patients in China, a model of holistic AF care supported by mobile health technology reduced the short-term risks of rehospitalization and clinical adverse events. There were several limitations, including 8% of patients lost to follow-up and younger and healthier patients in the active compared with the usual-care arm.^29^ Yet another cluster-randomized controlled trial with 1732 patients across 70 centers in 6 countries found that a program for the education of health care professionals could improve patient-level adherence to clinical practice guidelines on AF, whereas it had no significant effect on stroke prevention.^30^

The ABC-AF risk scores have consistently been shown to be well calibrated and superior to traditional risk scores based on clinical variables.^10–15,31^ Thus, although the ABC-AF–guided multifactorial study intervention evaluated in this study was not shown to be beneficial, the use of more precise and quantifiable risk scores may still be considered to identify patients with AF at high risk of stroke and/or bleeding. Future study approaches may include identification of patients suitable for novel, safer, or more effective OAC drugs or a left atrial appendage occlusion device used in combination with OAC or as an alternative to OAC treatment. In addition to improving the risk prediction for stroke and bleeding, the cardiovascular biomarkers included in the ABC-AF risk scores are also strongly associated with fatal events, the most common cardiovascular event in anticoagulated patients with AF.^32^ These fatal events are usually associated with heart failure death or sudden death, which should be targeted in future clinical studies of AF.

### Limitations

The main limitation was that the study was underpowered, mainly because of the premature termination of recruitment because of a safety concern. In addition, the recruited patients had lower-than-aimed-for risk profiles, a higher rate of prestudy OAC treatment, modest differences in postrandomization treatments, and lower-than-anticipated event rates.

### Conclusions

In patients with AF, an individually tailored multidimensional risk-based treatment strategy, based on the ABC-AF-stroke and ABC-AF-bleeding risk scores, was not associated with any improvements in clinical outcomes compared with usual guideline-based care. There were no interactions between risk classification and the effects of the multidimensional strategy on outcomes. The results emphasize the need for prospective testing of the use of risk stratification and precision medicine tools in different clinical settings before their implementation in routine care.

## Article Information

### Acknowledgments

The authors thank the data monitoring committee members Bertil Olsson (chair), Cecila Linde, and the late Hans Wedel. The authors also thank the staff at the Uppsala Clinical Research Center, Uppsala University, including Project Managers; Biostatisticians; Data Manager; Publications Manager; and Mats Lind, for designing the visualization of ABC-AF risk score results and aligned recommendations. The authors acknowledge Björn Berglund, patient representative in the study steering committee; all cardiologists and research nurses involved in the study in Sweden; and all participating patients. They also express gratitude to Agneta Siegbahn (chair) and all collaborators in the ABC risk scores project at Uppsala University.

### Sources of Funding

Funding was provided by grants from the Swedish Research Council (Dnr 2018-00894), the Swedish Heart-Lung Foundation (Dnr 2017-0829), and the Swedish Foundation for Strategic Research (Dnr RB13-0197; ABC risk scores project) and Roche Diagnostics, which, in addition, supplied instruments, biochemical assays, and laboratory support.

### Disclosures

Dr Oldgren reports institutional research grants from Amgen, AstraZeneca, Bayer, Novo Nordisk, and Roche Diagnostics; advisory board fees from Regeneron; and lecture fees from Pfizer, paid to his institution. Dr Hijazi is employed by Thermo Fisher Scientific. During the ABC-AF study, he reports research support from The Swedish Society for Medical Research (S17-0133), The Swedish Heart-Lung Foundation (20200722), and Uppsala University Hospital, Sweden; lecture/consulting fees from Bayer, Bristol-Myers Squibb, Pfizer, and Roche Diagnostics; and participation on data safety monitoring boards for Bristol-Myers Squibb and Pfizer. Dr Björkenheim reports speaker honoraria from Abbott, Bayer, and Medtronic. Dr Ravn-Fischer reports lecture fees from Amarin, Amgen, AstraZeneca, Bayer, Boehringer Ingelheim, Bristol-Myers Squibb, Johnson and Johnson, Lilly, Novartis, Novo Nordisk, Orion Pharma, Organon, Sanofi, and Pfizer; she is an advisory board member for Amarin, Amgen, AstraZeneca, Novartis, and Sanofi and a board member of RIKS-HIA (unpaid). Dr Själander reports lecture fees from Bayer, Bristol-Myers Squibb, and Pfizer and is chair of the Swedish Society on Thrombosis and Haemostasis. Dr Wallentin reports receiving grants and equipment and assays from Roche Diagnostics and holds 2 patents on GDF-15 (Nos. EP2047275 B1 and US8951742 B2).

### Supplemental Material

CONSORT Checklist

Figures S1 and S2

## Supplementary Material
