# Supplementary material for: Biomarker-Based ABC-AF Risk Scores for Personalized Treatment to Reduce Stroke or Death in Atrial Fibrillation: A Registry-Based, Multicenter, Randomized, Controlled Study
Source: Circulation. 2025 Aug 30;152(21):1457–69. doi: 10.1161/CIRCULATIONAHA.125.076725 (PMC12643572; doi:10.1161/CIRCULATIONAHA.125.076725)
Supplement: Supplementary file 1 [file cir-152-1457-s001.pdf]

SUPPLEMENTAL MATERIAL

Biomarker-based ABC-AF risk scores for personalized treatment to reduce stroke or death **in atrial fibrillation** – a registry-based, **multicent**er, randomized, controlled study

Oldgren, et al.

Table of contents

CONSORT Checklist...2
Figure S1. CONSORT flow chart for the ABC-AF study...4
Supplemental methods..5
Figure S2. ABC-AF risk score categories and recommendations..5
Study Steering Committee 7
Data Monitoring Committee 7
Principal Investigators..7

CONSORT Checklist

Section/topic	No	CONSORT 2025 checklist item description	Reported on page no.
**Title and abstract**			
Title and structured abstract	1a	Identification as a randomized trial	1
	1b	Structured summary of the trial design, methods, results, and conclusions	3-4
**Open science**			
Trial registration	2	Name of trial registry, identifying number (with URL) and date of registration	4
Protocol and statistical analysis plan	3	Where the trial protocol and statistical analysis plan can be accessed	- (Ref17)
Data sharing	4	Where and how the individual de-identified participant data (including data dictionary), statistical code and any other materials can be accessed	8
Funding and conflicts of interest	5a	Sources of funding and other support (eg, supply of drugs), and role of funders in the design, conduct, analysis and reporting of the trial	13 and 22
	5b	Financial and other conflicts of interest of the manuscript authors	22-23
**Introduction**			
Background and rationale	6	Scientific background and rationale	7-8
Objectives	7	Specific objectives related to benefits and harms	8
**Methods**			
Patient and public involvement	8	Details of patient or public involvement in the design, conduct and reporting of the trial	Supplement p7
Trial design	9	Description of trial design including type of trial (eg, parallel group, crossover), allocation ratio, and framework (eg, superiority, equivalence, non-inferiority, exploratory)	8
Changes to trial protocol	10	Important changes to the trial after it commenced including any outcomes or analyses that were not prespecified, with reason	13
Trial setting	11	Settings (eg, community, hospital) and locations (eg, countries, sites) where the trial was conducted	13
Eligibility criteria	12a	Eligibility criteria for participants	8-9
	12b	If applicable, eligibility criteria for sites and for individuals delivering the interventions (eg, surgeons, physiotherapists)	-
Intervention and comparator	13	Intervention and comparator with sufficient details to allow replication. If relevant, where additional materials describing the intervention and comparator (eg, intervention manual) can be accessed	9-10 and Supplement
Outcomes	14	Prespecified primary and secondary outcomes, including the specific measurement variable (eg, systolic blood pressure), analysis metric (eg, change from baseline, final value, time to event), method of aggregation (eg, median, proportion), and time point for each outcome	10
Harms	15	How harms were defined and assessed (eg, systematically, non-systematically)	10
Sample size	16a	How sample size was determined, including all assumptions supporting the sample size calculation	11
	16b	Explanation of any interim analyses and stopping guidelines	-
Randomisation:			
Sequence generation	17a	Who generated the random allocation sequence and the method used	9
	17b	Type of randomization and details of any restriction (eg, stratification, blocking and block size)	9
Allocation concealment mechanism	18	Mechanism used to implement the random allocation sequence (eg, central computer/telephone; sequentially numbered, opaque, sealed containers), describing any steps to conceal the sequence until interventions were assigned	9
Implementation	19	Whether the personnel who enrolled and those who assigned participants to the interventions had access to the random allocation sequence	-
Blinding	20a	Who was blinded after assignment to interventions (eg, participants, care providers, outcome assessors, data analysts)	8
	20b	If blinded, how blinding was achieved and description of the similarity of interventions	-

Statistical methods	21a	Statistical methods used to compare groups for primary and secondary outcomes, including harms	11-12
	21b	Definition of who is included in each analysis (eg, all randomized participants), and in which group	11
	21c	How missing data were handled in the analysis	-
	21d	Methods for any additional analyses (eg, subgroup and sensitivity analyses), distinguishing prespecified from post hoc	12
**Results**			
Participant flow, including flow diagram	22a	For each group, the numbers of participants who were randomly assigned, received intended intervention, and were analyzed for the primary outcome	13 and Figure S1
	22b	For each group, losses and exclusions after randomization, together with reasons	Figure S1
Recruitment	23a	Dates defining the periods of recruitment and follow-up for outcomes of benefits and harms	13
	23b	If relevant, why the trial ended or was stopped	13
Intervention and comparator delivery	24a	Intervention and comparator as they were actually administered (eg, where appropriate, who delivered the intervention/comparator, how participants adhered, whether they were delivered as intended (fidelity))	14-15
	24b	Concomitant care received during the trial for each group	14-15 (Table 2)
Baseline data	25	A table showing baseline demographic and clinical characteristics for each group	Table 1
Numbers analysed, outcomes and estimation	26	For each primary and secondary outcome, by group:<br>● the number of participants included in the analysis<br>● the number of participants with available data at the outcome time point<br>● result for each group, and the estimated effect size and its precision (such as 95% confidence interval)<br>● for binary outcomes, presentation of both absolute and relative effect size	15 and Table 3
Harms	27	All harms or unintended events in each group	15
Ancillary analyses	28	Any other analyses performed, including subgroup and sensitivity analyses, distinguishing pre-specified from post hoc	15-16
**Discussion**			
Interpretation	29	Interpretation consistent with results, balancing benefits and harms, and considering other relevant evidence	16-21
Limitations	30	Trial limitations, addressing sources of potential bias, imprecision, generalizability, and, if relevant, multiplicity of analyses	20

Figure S1. CONSORT flow chart for the ABC-AF study.

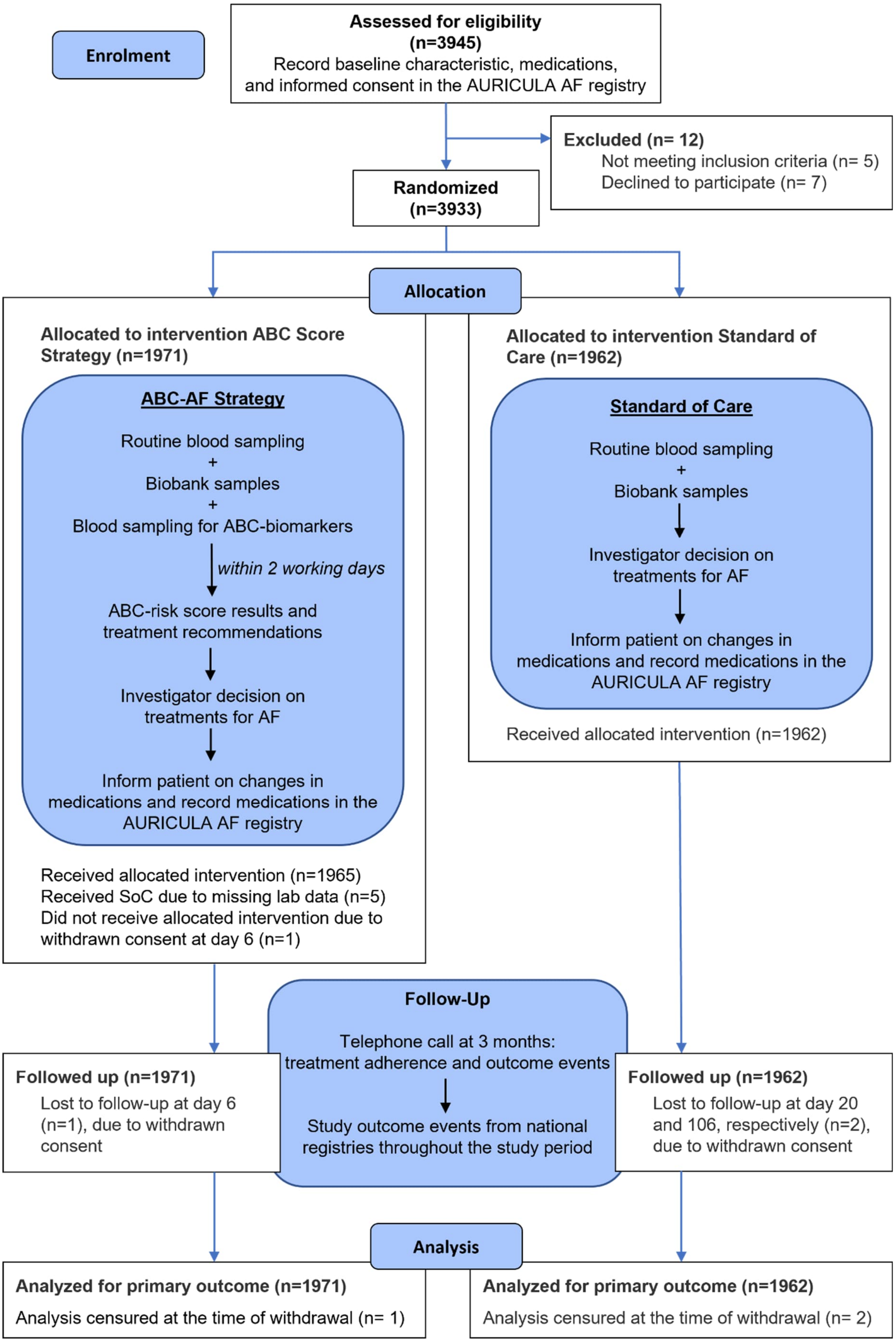

Supplemental methods

Summary of ABC-AF stroke and bleeding risk categories, recommended treatments and interventions, and numbers of patients in each of the six ABC-AF risk score categories.

Figure S2. ABC-AF risk score categories and recommendations

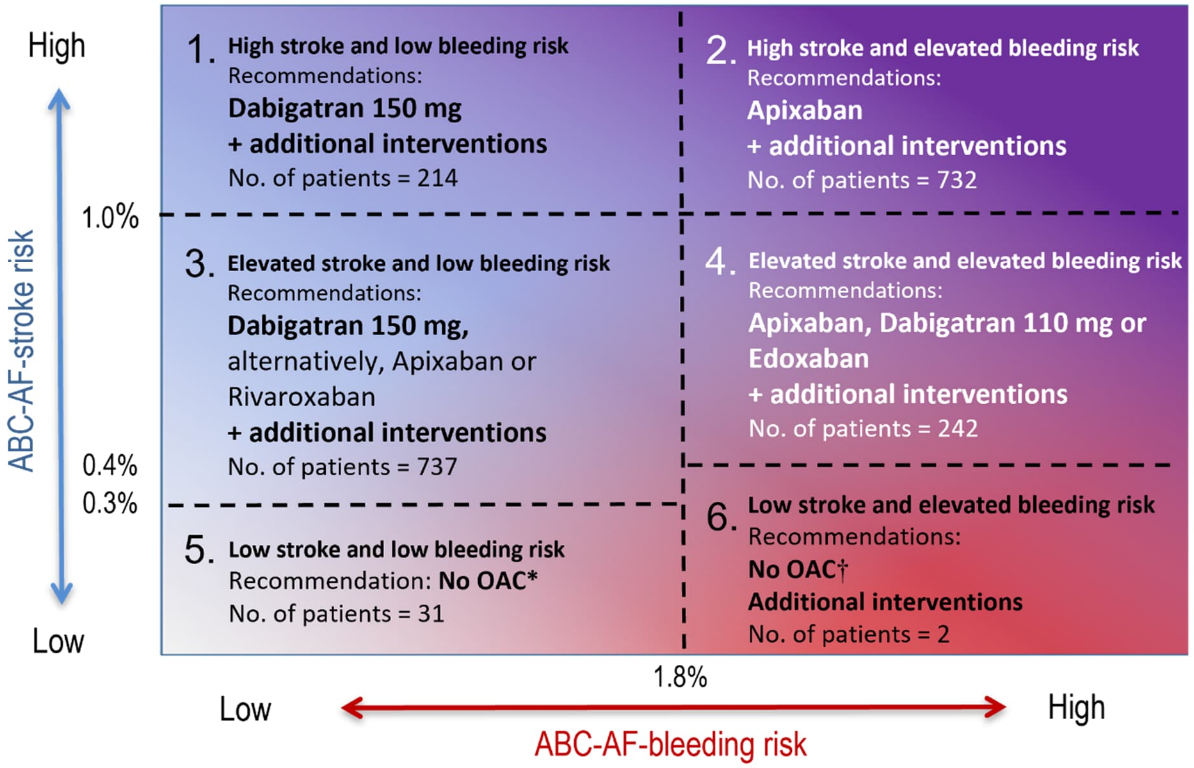

*Patients with a prior stroke in ABC-AF risk group 5 automatically received a recommendation for DOAC according to risk group 3.
†Patients with a prior stroke in ABC-AF risk group 6 automatically received a recommendation for DOAC according to risk group 4.

Of note, only one out of six different sets of recommendations, based on the balance between stroke and bleeding risk for the individual patient, was presented to the investigator for each patient randomized to the ABC-AF strategy arm.[17]

Additional interventions

PATIENTS WITH ELEVATED OR HIGH STROKE RISK BASED ON ABC-AF-STROKE RISK SCORE (risk categories 1, 2, 3 or 4 above)

- Optimize cardiovascular treatment
  - Initiate ACE-inhibitor (angiotensin receptor blocker) or mineralocorticoid receptor antagonists
- Investigate heart failure
  - Echocardiographic assessment, if not performed
- Optimize LDL-cholesterol
  - Initiate/increase dosages of statin therapy if LDL >1,8 mmol/L
  - In particular if prior stroke/TIA, coronary artery disease, or peripheral arterial disease
- Optimize heart rate
  - Target heart rate at rest <80 beats per minute with beta-blockers

- Consider cardioversion to sinus rhythm if feasible
• Investigate/optimize underlying diabetes
  - If diabetes is established, consider newer treatments which also lowers the risk of cardiovascular events
  - Consider referral to endocrinologist

ELEVATED BLEEDING RISK BASED ON ABC-AF-BLEEDING RISK SCORE (risk categories 2, 4 and 6 above)

• Check hemoglobin, if hemoglobin <120 g/L in women, <130 g/L in men:
  - Investigate anemia
  - Treat causes of anemia
• Initiate treatment with a proton pump inhibitor
• Discontinue NSAID treatment if present and possible
• Discontinue antiplatelet therapy if present and possible
• Consider malignant disease
  - In particular in the presence of other signs, e.g. anemia, hematuria, or feces hemoglobin

***Rationale for risk categories and associated interventions in the** ABC-AF strategy arm*

The rationale for the six stroke and bleeding risk categories has been published.[17] In brief, the risk categories were based on commonly used cut-off levels for stroke prevention, i.e. an annual stroke risk threshold of 0.3-0.4% with OAC treatment (corresponding to 0.9-1.0% without OAC), as well as a threshold to identify patients at high stroke risk of at 1.0% with OAC and 3.0% without OAC. The threshold for elevated bleeding risk was defined as annual bleeding risk of 1.8% during OAC treatment. This cut-off was slightly lower than the more commonly used 2.0% to provide a safety margin in the study — recognizing the human body's biological systems and that bleeding risk is a continuum rather than a dichotomous variable. There are no randomized studies directly comparing the individual DOACs, therefore the recommendations on specific types of DOAC for the individual patient in the ABC-AF strategy arm was based on the results of the four pivotal trials comparing apixaban, dabigatran, edoxaban, and rivaroxaban, respectively, versus warfarin, as well as large real-world observational studies. The recommendations to consider screening for and optimizing treatment of heart failure, dyslipidemia, and diabetes, including use of risk lowering therapies, such as Renin-Angiotensin-Aldosterone System (RAAS) blockade, or statins, as well as mitigating bleeding risks through measures such as addressing anemia, individualizing gastric protection, and avoiding NSAIDs and concomitant platelet inhibition therapy, are consistent with the holistic, integrated care approach advocated in AF guidelines.[4]

Of note, only one out of six different sets of recommendations, selected according to the balance between ABC-AF stroke and bleeding risk, is presented to the investigator for each patient in the ABC-AF strategy arm, available in online appendix to the ABC-AF design manuscript.[17]

STUDY STEERING COMMITTEE

The study steering committee comprised Jonas Oldgren (Coordinating investigator), Ziad Hijazi (Co-coordinating investigator), Håkan Arheden, Björn Berglund (Patient representative), Anna Björkenheim, Viveka Frykman, Magnus Janzon, Annica Ravn-Fischer, Anders Själander, Torbjörn Åkerfeldt, and Lars Wallentin (Chair).

DATA MONITORING COMMITTEE

The Data Monitoring Committee comprised Bertil S Olsson, MD, PhD (Chair), Cecilia Linde, MD, PhD, and Hans Wedel, PhD.

PRINCIPAL INVESTIGATORS

Dr. Ali Razooqi (95), Västmanlands sjukhus, Köping; Dr. Anders Månsson (29), Helsingborgs lasarett, Helsingborg; Dr. Anna Björkenheim (609), Universitetssjukhuset Örebro, Örebro; Dr. Annica Ravn-Fischer (24), Sahlgrenska universitetssjukhuset, Göteborg; Dr. Azad Dilan (341), Västmanlands sjukhus, Sala; Dr. Birgitta Johansson (12), Östra sjukhuset, Göteborg; Dr. Carl-Johan Lindholm (142), Clemenstorgets hjärtmottagning, Lund; Dr. Carlos Valladares (78), Hjärthälsan, Linköping; Dr. Charis Karakosta Papachristou (106), Karlstad lasarett, Karlstad; Dr. Christos Milonas (159), CityHeart, Stockholm; Dr. Cornelia Varadan (103), Hallands sjukhus, Halmstad; Dr. David Olsson (46), Länssjukhuset i Kalmar; Dr. Emma Sandgren (51), Hallands sjukhus, Varberg; Dr. Faris Al-Khalili (173), Sophiahemmet, Stockholm; Dr. Filip Jacobsson (20), Göteborgs hjärtmottagning, Göteborg; Dr. Georgios Mourtzinis (95), Mölndals sjukhus, Mölndal; Dr. Henrik Olsson (10), Norra Älvsborgs lasarett, Trollhättan; Dr. Isa Jalali (43), Lasarettet i Enköping, Enköping; Dr. Jon Erik Jonsson (92), Södersjukhuset, Stockholm; Dr. Jonas Andersson (1), Skellefteå sjukhus, Skellefteå; Dr. Kaveh Azizi (167), Västmanlands

sjukhus, Västerås; Dr. Kaveh Keshavaraz (5), Mälarsjukhuset, Eskilstuna; Dr. Lars Karlsson (199), Linköpings universitetssjukhus, Linköping; Dr. Martina Walter (29), Skaraborgs sjukhus, Lidköping; Dr. Michael Guggi (47), Falu lasarett, Falun; Dr. Oras Al-Khamisi (9), Karlskoga lasarett, Karlskoga; Dr. Panagiotis Mallios (51), Vrinnevisjukhuset, Norrköping; Dr. Regina Lindberg (42), Länsjukhuset Ryhov, Jönköping; Dr. Reza Nouri Mahdavi (17), Gävle lasarett, Gävle; Dr. Richard Müller-Brunotte (35), Stockholm Heart Center, Stockholm; Dr. Sara Själander (5), Sundsvalls sjukhus, Sundsvall; Dr. Stefan Lind (255), Karolinska universitetssjukhuset, Solna and Huddinge; Dr. Tord Juhlin (18), Skånes universitetssjukhus, Lund; Dr. Vasilios Marselos (192), Lasarettet i Motala; Dr. Veronika Högfeldt Mikelsen (15), Norrtälje sjukhus Tiohundra, Norrtälje; Dr. Viveka Frykman (330), Danderyds sjukhus, Danderyd; Dr. Ziad Hijazi (288), Akademiska sjukhuset, Uppsala.
